# Article processing charges and health research output in low-income countries: funding cuts, implications for health policy and system management

**DOI:** 10.3389/frhs.2025.1686682

**Published:** 2025-12-04

**Authors:** Stanley Chinedu Eneh, Francisca Ogochukwu Onukansi, Chidera Gabriel Obi, Collins Chibueze Anokwuru, Ogechi Vinaprisca Ikhuoria, Chioma Adaora Nwalieji, Moses Ifeatu Nwuzoh, Onyeka Chukwudalu Ekwebene, Ephraim Ikpongifono Udokang, Okoli Chukwudinma Chigozie, Ugonma Winnie Dozie, Temitope Olumuyiwa Ojo

**Affiliations:** 1 Department of Community Health, Obafemi Awolowo University, Ile-ife, Osun, Nigeria; 2 IVAN Research Institute, Enugu, Nigeria; 3 Youth in Research Hub, Enugu, Nigeria; 4 Department of Public Health, Federal University of Technology Owerri, Owerri, Nigeria; 5 Department of Parasitology and Entomology, Nnamdi Azikiwe University Awka, Awka, Anambra, Nigeria; 6 Department of Population & Reproductive Health, School of Public Health, University of Port Harcourt, Choba, Rivers, Nigeria; 7 University of Nigeria Teaching Hospital Ituku Ozalla, Enugu, Nigeria; 8 Depertment of Public Health, Sheffield Hallam University, Sheffield, United Kingdom; 9 Department of Pediatrics, Ryan White Center for Pediatric Infectious Disease and Global Health, Indiana University School of Medicine, Indianapolis, IN, United States; 10 Faculty of Pharmacy, University of Uyo, Uyo, Akwa Ibom, Nigeria; 11 FCT Medical Lab Services, Hospitals Management Board, Abuja, Nigeria

**Keywords:** article processing charges (APCs), open-access publishing, health research equity, low-income countries (LICs), research funding cuts, global knowledge inequality, diamond open access models

## Abstract

Article processing charges (APCs) pose a material barrier to the dissemination of health research from low income countries where recent funding cuts compound limited domestic financing and fragile health systems. Despite carrying a disproportionate share of global disease, these settings contribute under one percent of global research publications. This Perspective piece explores how APCs and funding cuts intersect to shape research output, summarises mitigation efforts and gaps, and proposes practical options for more equitable access to scholarly publishing. APCs are reported to shape venue choice for researchers in low income countries, while reduced external funding leaves fewer upstream resources to absorb costs. Country examples point to institutional and capacity pressures. Early career researchers often face disproportionate obstacles including slower progression and reduced competitiveness. Waiver policies and regional initiatives such as AJOL, SciELO South Africa and AfricArXiv offer partial relief, yet inconsistencies in eligibility, awareness and implementation persist with ethical implications. A rights and equity oriented response would include tiered APC models, automatic waivers linked to country income classification, ring fenced support for health research in low income settings, greater investment and independent evaluation of diamond open access platforms, and focused research on the effects of funding cuts on APCs and dissemination in low income contexts.

## Background

An article processing charge (APC) is a price occasionally placed on authors for the publication of an article in an academic journal, whether open access (hybrid or fully open) or entirely closed journals (1). These charges are often used to cover the cost of publishing the articles, from production (including editing and reviewing) to the necessary technical infrastructure (like the journal's website and management systems) and even marketing. Typically, the charges are covered by the author, their college or university, or the organization financing the research. For open access (OA) journals, APCs also contribute to making the research work available as open access (2).

APCs typically range from a few hundred to thousands of dollars, with an average of about $1,350 and even up to $3,900 in premium journals (3). Thus, for researchers in low-income countries, this can pose a significant barrier. In fact, this issue is closely linked to broader challenges in research for African scientists, particularly limited funding and inability to secure research grants (4). Furthermore, these challenges can manifest more prominently in countries with unfavorable conversion rates caused by weak economies. Consequently, researchers from low-income countries are often unable to cover APC, even when partial waivers and discounts are offered because of poor or lack of research funding. Indeed, APCs are allegedly a major barrier to the advancement of science in Africa (4, 5) and hinder equitable participation in the global research landscape. This review highlights the challenges that researchers in low-income countries face in covering these fees, and it aims to advocate for more inclusive and fairer publishing practices that eliminate this barrier for researchers in these areas.

The rise of Open Access (OA**)** Publishing marks a transformative shift in scholarly communication, addressing the limitations of traditional publishing models (6). Before the digital era, publishers and academic societies controlled the dissemination of research, but the transition to electronic formats introduced access and preservation challenges, especially as much of the scholarly record existed solely in digital form (6). These issues were compounded by the increasing volume and cost of academic journals, making it difficult for libraries—particularly in developing countries to afford subscriptions (7). This challenge has systematically impacted the sharing of health and clinical findings from low-income countries (LICs), where research finds it difficult to share evidence findings a result of high cost of APC charges leading to challenges in health policy making and clinical practice (8). Over time, OA has gained significant support through initiatives, policies, and funding mandates across disciplines, creating a more inclusive and equitable research ecosystem (6). By democratizing knowledge, OA empowers researchers to share findings widely, ensuring that financial constraints no longer hinder access to critical information, especially evidence-based practices. This can lead to limited opportunities for publishing findings in reputable journals to enhance evidence base practice in health care settings. Thus, this paper focused on how article processing charges and recent reductions or cuts in external health financing intersect to shape research output, and access to evidence in low-income countries, and to identify practical mitigation.

## Global disparities in research funding and resources

A nation's innovative capacity is driven not only by development of a workforce equipped to perform technologically advanced activities but also by its investments in research and development (R&D). According to the U.S. and global research and development (9), Global R&D expenditures increased significantly from $726 billion in 2000 to an estimated $2.4 trillion in 2019, driven by growth in middle-income countries, particularly in East and South Asia. While the United States remained the largest spender in 2019, accounting for $656 billion or 27% of global R&D, its share declined as countries like China experienced rapid growth, contributing 29% to global R&D expansion during this period (9). Together, the United States and China performed about half of the world's R&D, with other notable contributors including Japan (7%), Germany (6%), South Korea (4%) France (3%), India (3%), and the United Kingdom (2%). Smaller economies like Israel, South Korea, and Taiwan exhibited higher R&D intensity than the United States, with South Korea and China experiencing the fastest growth in R&D intensity from 2000 to 2019 (9) (see Table 1).

**Table 1:** Global disparities in research funding.

Country/Region	R&D expenditure (% of GDP)	Research funding received (US$)	Notable observations
United States (9)	2.81%	$656 billion (2019)	Accounts for 27% of global R&D expenditure
China (9)	2.11%	$526 billion (2019)	Represents 22% of global R&D expenditure.
Israel (14)	6%	-	Highest R&D expenditure as a percentage of GDP
South Korea (14)	5.2%	-	Significant investment in R&D is related to GDP.
Argentina (15)	0.52%	-	
Uruguay (16)	0.40%	-	
Mali (17)	0.29%	-	
Armenia (18)	0.22%	-	
Iraq (19)	0.04%	-	
Guatemala (20)	0.02%	-	
Egypt (21)	0.72%	-	
Upper-Middle-Income Countries (UMICs) (22–24)	-	$193 million as of 2020	Received 0.5% of global health research grants.
Low-Income Countries (LICs) (22, 23)	-	$85 million as of 2020	Received only 0.2% of global health research grants.
Lower-Middle-Income Countries (LMICs) (22, 23)	-		Received 0.5% of global health research grants.

- = Data not specified.

Global health research funding continues to exhibit stark disparities, with high-income countries (HICs) dominating research output and funding allocation. Although HICs account for only 16% of the global population and 10% of the disease burden, they produce approximately 80% of research publications, focusing primarily on diseases affecting their populations (10). Upper-middle-income countries (UMICs) contribute 17% of research publications, while low-income countries (LICs) produce a mere 0.6%. These trends underscore the persistence of the “10/90 gap,” where less than 10% of global health research resources address the health challenges of 90% of the world's population living in low- and middle-income countries (10). Considering the current uncertainty of withdrawal funds from the United States (11), African and other LICs may finds it difficult to share health related finding as result of APC charges and poor health fundings.

Consequentially, funding inequities are further reflected in grant allocations. In 2020, LICs received only 0.2% (US$ 85 million) of the US$ 37 billion in grants from the world's largest health research funders, with LMICs and UMICs receiving 0.5% each (12). These figures are consistent with 2016 data, highlighting the lack of progress in addressing these disparities (13). Despite ongoing concerns and recommendations, the inequitable distribution of resources remains a critical challenge in global health research.

## Health funding cuts in low-income countries

External health financing cuts are squeezing health sectors and research budgets across low-income countries, compounding barriers created by APCs. Recent OECD data and tracking by the Institute for Health Metrics and Evaluation indicate a 7.1% fall in official development assistance in 2024, a further 9%–17% decline expected in 2025, and an overall decrease of roughly one fifth between 2024 and 2025, largely reflecting reductions in United States funding and other donors' reprioritisation towards domestic pressures, humanitarian emergencies and geopolitical commitments (24, 25). Because many low-income countries remain structurally dependent on external funds for both health services and research, existing inequalities are likely to widen (26, 27). Uganda's National Health Accounts, for example, show that 46% of total health expenditure in 2020/21 was financed by external donors (28). When such funding withdraws or contracts, services, ongoing studies and publication plans are disrupted unless domestic resources are mobilised quickly (28), a condition that rarely holds for sustained research support.

Similar pressures are evident in Nigeria. Programmes for HIV, tuberculosis, sexual and reproductive health and malaria that previously benefited from international support were affected when the Global Fund allocation was cut by 11%, reducing grants by about $970 million in 2025 (29, 30). Although the 2025 health budget reportedly rose by 59.53% to $2.56 trillion, its dollar value fell by 16.45% due to currency depreciation, limiting the ability to offset lost external funds and potentially increasing the real cost of APCs and other research inputs (30–32).

Taken together, funding cut impose a double pressure on national budgets contract while funding for health research and publication also diminishes (33). This combination not only slows progress on disease control and essential health interventions but also places additional financial burdens on researchers and institutions (6, 26, 35). The joint effect of funding cuts and APC related costs therefore threatens immediate health outcomes and undermines the longer-term capacity required to strengthen public health systems.

## The effects of funding cuts, article processing charges of health research in low-income countries

The prevalent Article Processing Charge (APC) model in open-access publishing imposes substantial financial burdens on researchers, particularly those from low-income countries resulting in low-income sharing and other consequences as highlighted.

### Reduced research output

Researchers from low-income countries face significant financial constraints due to deterring APCs in the face of meagre resources, hindering publications, particularly in critical areas like health research. Limited publication opportunities restrict knowledge dissemination, exacerbating the knowledge gap between low-income and high-income countries, which is harmful in addressing global health challenges. Reliance on institutional or personal funds further restricts publication capacity (34). This financial burden disproportionately affects researchers from low-income countries, who often lack institutional funding to cover APCs. As a result, many researchers are forced to abandon their plans to publish their research, leading to a significant reduction in the visibility of research from these countries, including essential health studies that could inform local and global health policies (34, 35). The consequences of this reduced visibility are far-reaching. Researchers from low-income countries are less likely to receive citations, which can impact their career advancement and funding opportunities. In health research, this means critical findings on local disease patterns or health interventions remain underrepresented, limiting their contribution to global health solutions. Furthermore, the lack of representation of researchers from low-income countries in the global scientific literature perpetuates the dominance of researchers from high-income countries, exacerbating the existing knowledge gap (36), and hindering equitable progress in global health research.

### Barriers for early-career researchers

The spread of Article Processing Charges (APCs) has created a significant barrier for early-career health researchers in low-income countries. APCs, which can range from $1,356 to $5,200, are a substantial financial burden for researchers who lack institutional funding or grants to cover these costs, particularly in health fields where high-impact journals often charge high fees (23). As a result, early-career health researchers in low-income countries face limited publishing opportunities, which can hinder their career advancement and visibility in the global health scientific community. The inability to publish health-related research due to APC barriers also limits opportunities for collaboration with international colleagues, participation in global health research networks, and access to new knowledge and technologies which are important for addressing local health challenges (22, 23).

Furthermore, the APC barrier can delay career progression for early-career health researchers in low-income countries. The lack of publications and limited collaboration opportunities can make it challenging for these researchers to secure tenure-track positions, grants, or promotions in health research institutions. This can ultimately contribute to brain drain, as early-career health researchers may seek opportunities in high-income countries where APCs are more manageable or where institutional funding is more readily available to support health research (38). The loss of health researchers further entrenches the global health inequalities as low-income countries face huge hurdles retaining health researchers.

### Institutional challenges without robust funding

Health institutions in low-income countries struggle to allocate APC funds, diverting resources from essential activities, and the limited publication output affects institutional reputation and global rankings. This reduced visibility hinders their ability to attract funding or partnerships for critical health research (22). However, Long-term health research program sustainability is compromised due to APC-related financial burdens. Health institutions in low-income countries face significant challenges in covering Article Processing Charges (APCs), which can range from $1,356 to $5,200. The lack of robust funding for APCs hinders the ability of health researchers from these institutions to publish their work in reputable journals. This can lead to reduced visibility, limited collaboration opportunities, and delayed career progression for health researchers (22, 37). The financial burden of APCs also forces health institutions to make difficult decisions about which health research to support, potentially stifling innovation and progress in critical areas. Furthermore, the APC barrier can worsen existing inequalities in the global scientific landscape, perpetuating the dominance of researchers from high-income countries (37). The APC model disproportionately affects health institutions in low-income countries, intensifying knowledge disparities. Alternative publishing models, funding initiatives, and institutional support are necessary for equitable research opportunities. Addressing these challenges will foster a more inclusive global health research community.

## Current mitigation strategies of article processing charges (APC)

Regional open-access publishing initiatives, particularly in low-income countries in Africa, cannot be discussed without acknowledging the Health Inter-Network Access to Research Initiative (HINARI) (38). Established in 2001 by the World Health Organization (WHO), HINARI facilitates open access to biomedical and health literature in low- and middle-income countries (39). It has been celebrated for its significant role in increasing access to scholarly literature, and a rise in scientific research and knowledge in these regions (41). However, notable regional open-access (OA) initiatives in Africa include the African Journal Online (AJOL) and SciELO South Africa (SciELO SA) (41). AJOL, established in 1998, provides an online presence and catalog for African journals (42). This non-profit publishing platform also known as diamond open access (AO) hosts over 500 African-published journals, about half of which are open access (30). Diamond Open access journals are mainly supported by their publishing institutions and professional bodies but AJOL itself relies primarily on grant funding (42). On the other hand, SciELO SA, part of the Brazilian SciELO initiative since 2009, hosts 81 South African journals (43). It is funded by the national government and managed by the Academy of Science of South Africa (ASSAf) (29). Both AJOL and SciELO operate on a diamond open-access model, allowing authors to publish without any fees (40–42). Other initiatives, such as the African preprint server AfricArxiv, aim to mitigate article processing fees and enhance access (44). AfricArxiv enables researchers to share diverse outputs, including preprints, accepted manuscripts, working papers, and presentations (44).

Top publishers often use waiver policies to promote open-access publishing (45). However, these waiver policies are inconsistent, as each publisher establishes its own rules. Most publishers adopt Research4Life categories which require eligibility to determine publication fee waivers while others may provide only discount (46) without considering the LIC's financial burden. This implies that publishers may choose to grant full waivers (Free access) to researchers from Group A countries or partial waivers (full access with yearly subscription) to those from Group B countries (47). For eligibility using Research4life categories, Group A countries must meet at least one of the following criteria: be listed as a United Nations Least Developed Country, have a Gross National Income (GNI) of $500 million or less, a GNI of $5 billion or less with a GNI per capita (GNIpc) of $10,000 or less, a GNI of $15 billion or less with a GNIpc of $3,000 or less, or a GNI of $200 billion or less where the Human Development Index (HDI) is 0.60 or less, or the GNIpc is $1,500 or less (7), Group B countries must meet at least one criterion: a GNIpc of $6,300 or less and a Healthy Life Expectancy (HALE) of 55 or less, a GNI of $1.5 billion or less, a GNI of $25 billion or less with a GNIpc of $10,000 or less, or a GNI of $300 billion or less where the HDI is 0.67 or less, or the GNIpc is $6,300 or less (7). Eligible institutions include universities, research centers, teaching hospitals, government offices, and NGOs (48).

## Gaps and limitations of the APC approach amid funding cuts

Recent funding cuts and projected reductions in external financing may amplify the structural weaknesses of the article processing charge (APC) model (4, 26, 49). APC waiver programmes are often non public, inconsistent, and require burdensome justification (50). The APC model also rests on authors or their institutions having funds available to meet publication costs (51, 52). This leaves researchers in low income countries, especially those at institutions with limited support, to shoulder APCs personally despite being eligible for a waiver (4, 53).

Furthermore, cuts to official development assistance and development assistance for health (ODA/DAH) compress institutional and grant budgets (26, 49), which in turn may reduce the resources available to cover APCs for researchers in low income contexts. APCs in medical journals can be around US$4,600, a sum that may exceed a full year's salary in many low income settings (54). Faced with these constraints, some researchers may shift towards lower cost journals with weaker peer review, undermining ethical implications, dissemination, visibility, and equity in scholarship (4, 55). Evidence from African settings shows that unaffordable APCs can push authors, particularly early career researchers, towards predatory journals (56, 57).

Overall, the APC approach lacks resilience when funding contracts, especially where government research budgets in low income countries depend heavily on donors (58–60). The gap in equitable access widens as fiscal constraints associated with funding cuts intensify (26, 49, 61). Addressing these limitations requires not only rethinking APC models but also embedding funding stabilisation mechanisms for researchers in low and middle income countries (4, 55, 62).

Yet the specific influence of recent funding cuts on APCs and research output remains insufficiently theorised and empirically examined within current publishing and policy frameworks (26, 50). Existing studies on the effectiveness of APCs are mostly descriptive and have primarily focused on barriers to research output and dissemination (50, 63, 64)). Secondly, APC policy design trade offs are largely theorised rather than empirically tested, and studies evaluating interventions and their impact on equity remains limited (65, 66).

## Suggestions and a call for reconsideration

The gaps identified in this study suggest that recent funding cuts and inconsistent APC waiver practices have deepened inequities in global knowledge dissemination. These pressures particularly affect researchers in low and lower middle income countries (LICs and LMICs), whose institutions often lack the financial resilience to absorb publication costs when funding cuts or external funding contracts [4,26,49). To address the interlinked effects of funding cuts on APCs, we propose the following suggestions and considerations for publishing and funding frameworks, and for future research.

First, a tiered or progressive APC model could be considered, aligning fees with countries' income classifications and institutional funding capacity. Under this approach, authors from wealthier economies would continue to pay standard APCs, while those from LICs would qualify for partial or full waivers. Transparent implementation and periodic review especially at this time of funding cut are critical to avoid reinforcing existing inequities (50, 58).

Second, automatic and publicly available waiver policies should replace current discretionary or application based systems. Anchoring waivers in World Bank income classifications could reduce bureaucratic barriers that disadvantage researchers temporarily affiliated with high income institutions (51, 52).

Third, dedicated funding mechanisms are needed to stabilise publication support amid funding cut. Funders and governments could earmark a small percentage of health or research budgets for dissemination costs, helping maintain publication continuity during ODA or DAH reductions (25, 49). This would also support sustainable diamond open access models that rely on institutions rather than author funding.

**Fourth**, **topic targeted fee waivers** should prioritise research addressing major public health and development issues in LICs such as maternal and child health, infectious diseases, mental health, and climate resilience, where dissemination is most crucial for policy translation (67–74).

Lastly, given that the influence of funding cuts on APCs and research output remains insufficiently theorised and empirically examined (26, 49), future policy experiments should integrate evaluation research. Comparative and longitudinal studies measuring waiver uptake, publication diversity, and research visibility across funding cycles at the time of funding cuts would provide the evidence base needed to determine whether these measures effectively reduce inequities in global scholarship.

## Limitations and future directions

This paper draws on a diverse range of data sources, including published articles, policy documents, and reports. While we did not conduct a formal study following the IMRaD (Introduction, Methods, Results, and Discussion) structure or systematic review protocol, we acknowledge the importance of such approaches. We recommend that future research undertake a systematic or scoping review to better assess potential availability bias, particularly in the grey literature.

## Conclusion

In conclusion, the signals reviewed here suggest that APC requirements and reductions in external health financing may act together to constrain dissemination from resource limited settings with implications for representation in the literature and access to context relevant evidence. While these observations are descriptive and do not establish causality, they point to feasible adjustments that align with equity and integrity in research publishing. Progressive fee structures, automatically applied waivers, protected dissemination funds and careful support for diamond open access could reduce barriers without shifting costs onto individual researchers. Prioritising fee relief for work addressing major public health needs alongside clearer and publicly available waiver criteria may further strengthen policy translation. Future evaluations should track waiver uptake, submission and acceptance patterns and visibility outcomes across funding cycles with attention to early career authors and institutions in low and lower middle income countries.

## Data Availability

The original contributions presented in the study are included in the article/Supplementary Material, further inquiries can be directed to the corresponding author.
